# PAX2 Regulates ADAM10 Expression and Mediates Anchorage-Independent Cell Growth of Melanoma Cells

**DOI:** 10.1371/journal.pone.0022312

**Published:** 2011-08-18

**Authors:** Sophia Boyoung Lee, Kai Doberstein, Peter Baumgarten, Anja Wieland, Christopher Ungerer, Claudia Bürger, Katja Hardt, Wolf-Henning Boehncke, Josef Pfeilschifter, Daniela Mihic-Probst, Michel Mittelbronn, Paul Gutwein

**Affiliations:** 1 Pharmazentrum Frankfurt/ZAFES, University Hospital Goethe University Frankfurt, Frankfurt am Main, Germany; 2 Edinger Institute, Institute of Neurology, University of Frankfurt am Main, Frankfurt am Main, Germany; 3 Institute of Reconstructive Neurobiology, Life and Brain Center, University of Bonn and Hertie Foundation, Bonn, Germany; 4 Department of Dermatology, Clinic of the Goethe-University, Frankfurt, Germany; 5 Department of Dermatology, University Hospital Zurich, Zürich, Switzerland; Yale Medical School, United States of America

## Abstract

PAX transcription factors play an important role during development and carcinogenesis. In this study, we investigated PAX2 protein levels in melanocytes and melanoma cells by Western Blot and immunofluorescence analysis and characterized the role of PAX2 in the pathogenesis of melanoma. In vitro we found weak PAX2 protein expression in keratinocytes and melanocytes. Compared to melanocytes increased PAX2 protein levels were detectable in melanoma cell lines. Interestingly, in tissue sections of melanoma patients nuclear PAX2 expression strongly correlated with nuclear atypia and the degree of prominent nucleoli, indicating an association of PAX2 with a more atypical cellular phenotype. In addition, with chromatin immunoprecipitation assay, PAX2 overexpression and PAX2 siRNA we present compelling evidence that PAX2 can regulate ADAM10 expression, a metalloproteinase known to play important roles in melanoma metastasis. In human tissue samples we found co-expression of PAX2 and ADAM10 in melanocytes of benign nevi and in melanoma cells of patients with malignant melanoma. Importantly, the downregulation of PAX2 by specific siRNA inhibited the anchorage independent cell growth and decreased the migratory and invasive capacity of melanoma cells. Furthermore, the downregulation of PAX2 abrogated the chemoresistance of melanoma cells against cisplatin, indicating that PAX2 expression mediates cell survival and plays important roles during melanoma progression.

## Introduction

Malignant melanoma represents a substantial clinical challenge. It is one of the fastest-rising malignancies in the last several decades [1] and it is notorious for the propensity for metastasis and for the poor response to current therapeutic regimens. Understanding the molecular aberrations involved in the development and progression of malignant melanoma will be therefore essential for the development of new therapeutic strategies in the treatment of this aggressive and lethal skin disease.

Melanoma arises from melanocytes, which are neural crest-derived pigment cells that migrate to the subdermal layer of the skin and retina of the eye during embryogenesis. It has been reported that PAX3, one member of the PAX transcription factor family, plays an important role in melanocyte differentiation and proliferation [2]. The importance of PAX family members during development has been underscored by several loss-of function mutations that usually lead to a lack of the specific structures or organs where the PAX protein is normally expressed [3]. In addition, PAX genes are capable of acting as proto-oncogenes by transactivating promoters of target genes involved in the regulation of cell growth and apoptosis [4]. In humans, 9 PAX genes have been identified. All PAX genes commonly possess a paired domain, which can bind to DNA in sequence specific manner in order to function as transcription factors [4]. It is known that abnormal expression of PAX genes is associated with cancer development and progression. Abnormal expression levels of PAX genes through chromosomal translocations are found for example in thyroid cancer and acute lymphoblastic leukaemia [5], [6]. In melanoma patients PAX3 has been identified as a significant marker for melanoma staging [7], [8] and for the detection of circulating melanoma cells [7]. Importantly, the transfection of melanoma cells with antisense PAX3 oligonucleotides triggers cell death by inducing apoptosis [9], [10], highlighting the potential therapeutic option of targeting PAX3 in melanoma patients. In contrast to PAX3, no data exist about the expression and function of PAX2 in melanoma development and progression.

In the kidney PAX2 is critical for the survival of fetal collecting ducts and has a primary anti-apoptotic function in embryonic renal cells [11]. PAX2 expression is often restricted to embryogenesis and is down-regulated in adults but is reexpressed in several tumors like Wilms tumor [12], renal cell carcinoma [13], breast cancer [14] and karposi sarcoma [15]. Interestingly, we identified with the Transcriptional Element Search System (TESS) a published PAX binding site [16] in the promoter of ADAM10, a metalloproteinase which was significantly overexpressed in melanoma metastasis [17]. Therefore we wanted to characterize PAX2 expression in melanoma and investigate its role in the regulation of ADAM10. We found weak PAX2 expression in melanocytes and keratinocytes, but increased PAX2 levels in melanoma cell lines. Importantly, we present strong evidence, that PAX2 can regulate ADAM10 expression and that the downregulation of PAX2 inhibits the anchorage independent cell growth of melanoma cells. Furthermore we are able to demonstrate that PAX2 expression in melanoma cells is involved in the migration, invasion and cell survival of melanoma cells.

## Results

### PAX2 is differentially expressed in normal and neoplastic cells of the human skin

**Figure 1:**
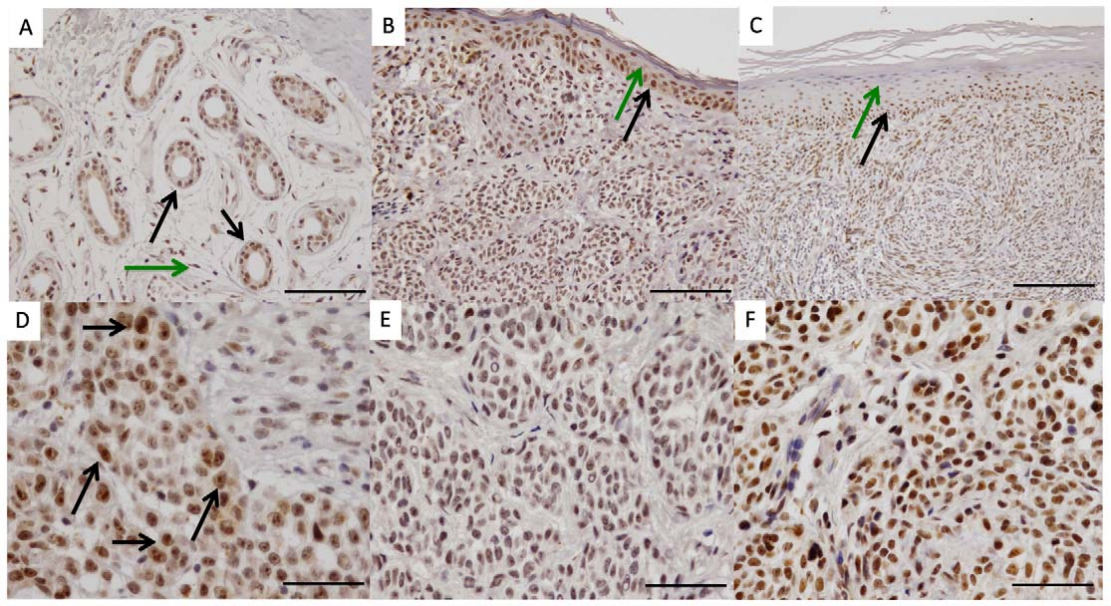
Immunohistochemical analysis of PAX2 expression in tissue sections of benign nevi and malignant melanoma. (**A**) In normal sweat glands, PAX2 is expressed in gland epithelial cells (black arrows) while intermingled stromal cells only show very weak or absent nuclear PAX2 expression (green arrow) Bar represent 100 µm. (**B, C**) Normal appearing epidermal cell layers adjacent to (**B,** bar represent 100 µm) nevi or (**C,** bar represent 200 µm) malignant melanoma show a differentially PAX2 expression with strongest PAX2 levels in germinal basal cell layers (black arrows) decreasing in higher differentiated keratinocytes and finally being absent in corneocytes (green arrows). (**D**) Malignant melanoma cells constantly exhibit a heterogeneous nuclear PAX2 expression. Strongest expression is observed in large atypical nuclei with prominent nucleoli (black arrows). Bar represent 50 µm. (**E, F**) PAX2 expression in intradermal nevi was heterogeneous and did not correlate with histological features (Original magnification: A–C: 20×; D–F: 40×). Bars represent 50 µm.

To determine the expression of PAX2 in human skin tissue we performed immunohistochemistry analysis on tissue sections of benign nevi and malignant melanoma. In normal skin, PAX2 was mainly expressed in nuclei of regenerating cells especially in epithelial cells of sweat gland and the germinal basal cell layers of the epidermis (Figure 1 A–C). In contrast, cells with low regenerative potential such as corneocytes of the most apical epidermal cell layer (Figure 1 B, C) or stromal cells intermingled between adnexal skin tissue (Figure 1 A) were mainly negative for PAX2. Both malignant melanomas (Figure 1 D) and intradermal nevi (Figure 1 E, F) show heterogeneous PAX2 expression levels. In malignant melanomas, nuclear PAX2 expression strongly correlated with nuclear atypia and the degree of prominent nucleoli (Figure 1 D) indicating an association of PAX2 with a more atypical cellular phenotype. In contrast, the heterogeneous PAX2 expression of intradermal naevi did not correlate with any obvious histological features. In particular, PAX2 expression in nevi was regionally regulated which was reflected in areas with absent or very weak nuclear PAX2 expression (Figure 1 E) as well as in regions with very prominent PAX2-positive nuclei (Figure 1 F).

### PAX2 regulates ADAM10 expression in melanoma cells

**Figure 2:**
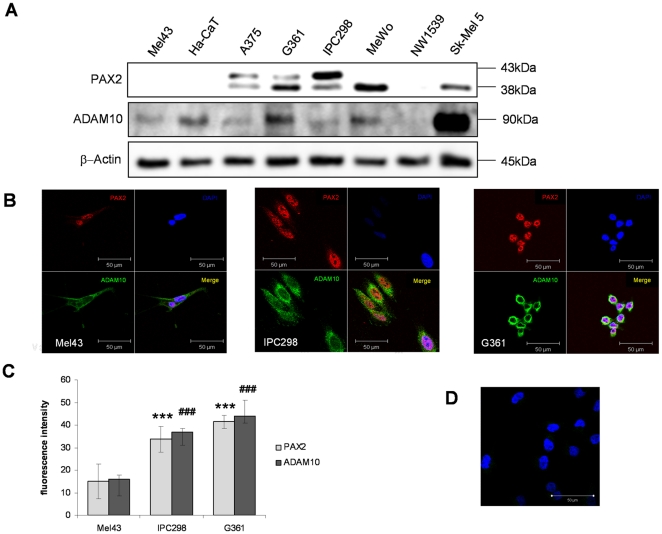
PAX2 and ADAM10 expression in melanocytes, keratinocytes and melanoma cells. (**A**) Western Blot analysis was performed to determine the PAX2 and ADAM10 expression in melanocytes (Mel43), keratinocytes and melanoma cells (A375, G361, IPC298, MeWo, NW1539 and SKMel5). Notably, 5 of 6 melanoma cell line show PAX2 and ADAM10 expression. β-Actin Western Blot analysis was performed to control equal protein loading. (**B**) Immunofluorescence staining of primary melanocytes Mel43 (left image) and the melanoma cell lines IPC298 (middle image) and G361 (right image) was performed to investigate the localisation of ADAM10 and PAX2. Cells were incubated with monoclonal ADAM10 and polyclonal PAX2 specific antibodies, followed by Alex488 coupled secondary antibodies (green) and Cy3 coupled secondary antibodies (red). The cells were stained with DAPI to visualize nuclei (blue). (**C**) The relative immunofluorescence intensity of ADAM10 and PAX2 expression in the melanocytes Mel43 and the melanoma celllines IPC298 and G631 were determined and depicted in a graph. ***P<0.001 PAX2 immunofluorescence intensity considered statistically significant compared to the PAX2 immunofluorescence intensity of melanocytes. **###**P<0.001 ADAM10 immunofluorescence intensity considered statistically significant compared to ADAM10 immunofluorescence intensity. (**D**) The specificity of ADAM10 and PAX2 immunofluorescence staining was controlled by using isotype specific control (control IgG) antibodies.

**Figure 3:**
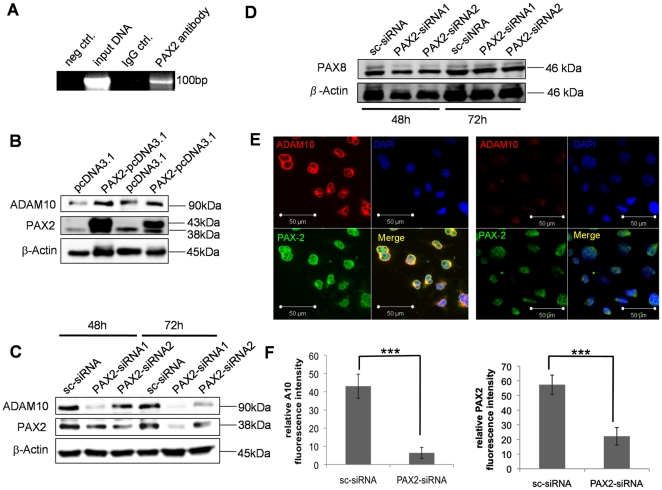
PAX2 regulates ADAM10 expression in melanoma cells. (**A**) Chromatin immunoprecipitation (ChIP) assay was performed with SKMel5 cells as described in material and methods. One representative experiment of three independently performed experiments is shown. (**B**) SKMel5 cells were transfected with pcDNA3.1 plasmid alone or with PAX2-pcDNA3.1 plasmid DNA. The expression of PAX2 and ADAM10 was determined by Western Blot analysis. β-actin was used to determine equal protein loading. (**C**) SkMel5 were transfected with 10 nM scrambled siRNA (sc-siRNA) or with 10 nM of two different PAX2-siRNAs (PAX2-siRNA1-2). 48 hours and 72 hours after the transfection, cells were lysed and the protein expression level of PAX2 and ADAM10 was investigated by Western Blot analysis. β-actin was used to determine equal protein loading. (**D**) SkMel5 were transfected with 10 nM scrambled siRNA (sc-siRNA) or with 10 nM of two different PAX2-siRNAs (PAX2-siRNA1-2). 48 hours and 72 hours after the transfection, cells were lysed and the protein expression level of PAX8 was investigated by Western Blot analysis. β-actin was used to determine equal protein loading. (**E**) Immunofluorescence analysis with ADAM10 and PAX2 specific antibodies were performed in sc-siRNA (left image) and PAX2- siRNA (right image) transfected SkMel-5 cells. ADAM10 expression was visualized by Cy3 coupled goat anti-mouse secondary antibodies, whereas PAX2 expression was detected with Alexa488 coupled goat anti-rabbit antibodies. (**F**) In the graphs the quantification of ADAM10 and PAX2 immunofluorescence intensity is shown. ***P<0.001 considered statistically significant compared to the sc-siRNA transfected SkMel5 cells.

To determine the expression levels of PAX2 and ADAM10 in keratinocytes, melanocytes and melanoma cells we performed Western Blot and immunofluorescence analysis. Although we could not detect any PAX2 expression in melanocytes and keratinocytes by Western Blot analysis (Fig. 2A), we found weak nuclear PAX2 expression in both cell lines by immunofluorescence analysis (Fig. 2B). In contrast to melanocytes and keratinocytes strong PAX2 expression was found in 5 of 6 melanoma cells (Fig. 2A). Comparing the PAX2 expression with ADAM10 expression, we found that all cell lines, which expressed PAX2 did also express ADAM10 (Fig. 2A). Only the melanoma cell line NW1539 expressed neither PAX2 nor ADAM10 (Fig. 2A). To determine the localisation of PAX2 and ADAM10 we performed immunofluorescence analysis in melanocytes and melanoma cells. Weak nuclear Pax2 expression was found in melanocytes compared to stronger nuclear PAX2 expression in the melanoma cell lines IPC298 and G631 (Fig. 2B). In contrast to nuclear PAX2, cytoplasmic and membranous ADAM10 expression was detectable in melanocytes and melanoma cells. Quantification of immunofluorescence staining in melanocytes revealed that weak PAX2 expression correlated with weak ADAM10 fluorescence intensity and stronger immunofluorescence staining of PAX2 in melanoma cells was accompanied by increased ADAM10 expression (Fig. 2C). To investigate, if PAX2 is involved in the regulation of ADAM10, we performed a chromatin immunoprecipitation (ChIP) assay. As shown in Fig. 3A the ADAM10 promoter fragment containing the PAX2 binding site, was only amplified in samples that were subsequently immunoprecipitated with PAX2 antibodies but not with control IgG antibodies. In addition to investigate if PAX2 can regulate ADAM10 protein expression, we overexpressed PAX2 in SKMel5 cells and determined ADAM10 expression by Western Blot analysis. As shown in Fig. 3B the overexpression of PAX2 led to an induction of ADAM10 protein levels. To further confirm that PAX2 is involved in the regulation of ADAM10 we downregulated PAX2 with two different siRNA (PAX2-siRNA1/2) in SkMel5 cells and performed Western Blot analysis. As shown in Fig. 3C by Western Blot analysis, the downregulation of PAX2 significantly decreased ADAM10 expression. To rule out off-targets effect by PAX2-siRNA, we confirmed that the downregulation of PAX2 did not reduce PAX8 levels (Fig. 3D). With immunofluorescence analysis we could confirm that the downregulation of PAX2 led to a decreased ADAM10 expression in melanoma cells (Fig. 3E and F). In summary, we can show that PAX2 can bind to the ADAM10 promoter and regulate ADAM10 protein levels in melanoma cells.

### PAX2 and ADAM10 are expressed in melanocytes of benign nevi and in melanoma cells of patients with malignant melanoma

**Figure 4:**
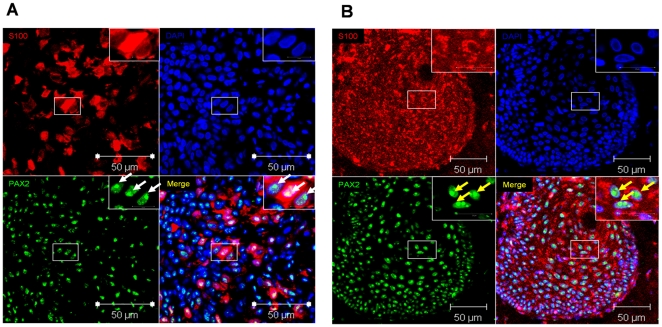
PAX2 is expressed in melanocytes of benign nevi and melanoma cells of patients with malignant melanoma. Tissue sections of benign nevi (**A**) and malignant melanoma (**B**) were investigated by double immunofluorescence analysis with S100 (melanocyte marker) and PAX2 specific antibodies. S100 expression was visualized by Cy3 coupled secondary antibodies (red) and PAX2 expression was detected with Alexa488 coupled goat anti-rabbit secondary antibodies (green). White arrows in the higher magnified insets indicate PAX2 expression in nucleoli of melanocytes of benign nevi (**A**), yellow arrows in the higher magnified insets specify PAX2 expression in nucleoli of melanoma cells (**B**).

**Figure 5:**
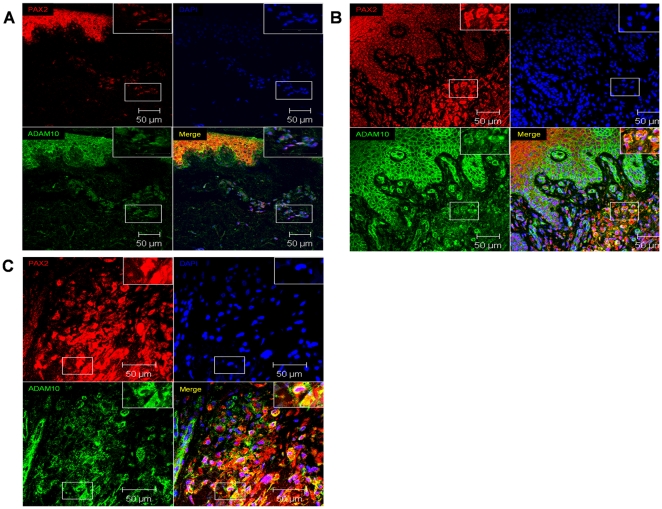
ADAM10 and PAX2 are co-expressed in melanocytes and melanoma cells in tissue sections of benign nevi and malignant melanoma. To determine if ADAM10 and PAX2 are co-expressed in melanocytes of benign nevi or in melanoma cells of patients with malignant melanoma, double immunofluorescence analysis on tissue sections has been performed. ADAM10 (**green**) and PAX2 (**red**) expression is detectable in melanocytes of benign nevi (**A** insets represent higher magnification of the single channels and the merged image of all 3 channels) and in melanoma cells of patients with malignant melanoma (**B** and **C** insets represent higher magnification of the single channels and the merged image of all 3 channels).

To investigate the expression of PAX2 in tissue sections of benign nevi and malignant melanoma we performed double immunofluorescence analysis. As shown in Fig. 4 PAX2 expression was detectable in S100 positive melanocytes of benign nevi (Fig. 4A) and in S100 positive melanoma cells (Fig. 4B). Importantly, PAX2 expression was visible in nucleoli of melanocytes (Fig. 4A, white arrows in the insets of PAX2 and merged images) and melanoma cells (Fig. 3B, yellow arrows in the insets of PAX2 and merged images). Notably, in melanoma patients larger PAX2 expressing nucleoli were detectable (Fig. 3B, merged image, yellow arrows). To further determine if ADAM10 and PAX2 are co-expressed in melanocytes and melanoma cells in situ double immunoflourescence analysis on tissue sections were performed. ADAM10 and PAX2 were co-expressed in melanocytes of benign nevi (Fig. 5A, higher magnification of melanocytes expressing ADAM10 and PAX2 is depicted in the inset) and in melanoma cells of patients with malignant melanoma (Fig. 5B+C, higher magnifications of melanoma cells expressing ADAM10 and PAX2 are depicted in the insets). In summary we can conclude, that melanocytes and melanoma cells in situ co-express ADAM10 and PAX2.

### Knockdown of PAX2 by siRNA inhibits anchorage dependent and independent cell growth of melanoma cells

**Figure 6:**
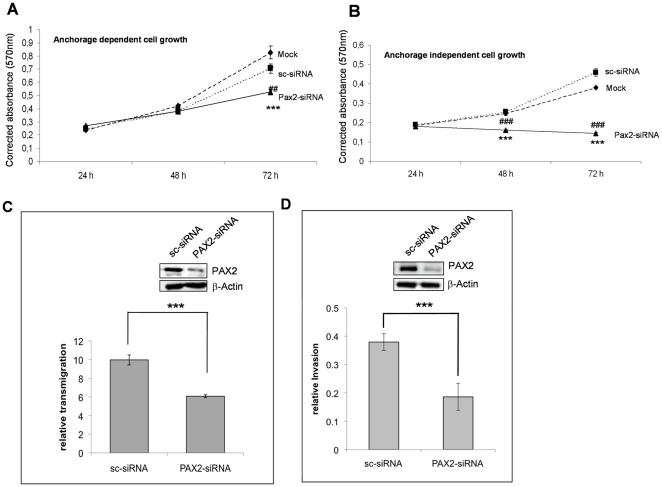
Downregulation of PAX2 decreases the proliferation, migration and invasion of melanoma cells. Anchorage-dependent (**A**) and anchorage-independent (**B**) cell growth was investigated by using a MTT proliferation assay. Twenty-four hours after siRNA transfection, SkMel5 cells treated with transfection reagents alone (mock) or transfected with scrambled siRNA (sc-siRNA) or PAX2-specific siRNAs were seeded into uncoated anchorage dependent cell growth) or polyHEME coated (anchorage independent cell growth) 96 well plates and cell growth was measured 24, 48 and 72 hours later using a MTT-assay. 3 independent experiments have been performed and statistical analysis has been performed using Anova post-hoc analysis. ***P<0.001 considered statistically significant compared to control transfected cells (Mock). **###**P<0.001 considered statistically significant compared to scrambled-siRNA transfected cells, *P<0.01 considered statistically significant compared to scrambled-siRNA transfected cells. (**C**) Migration assay of SkMel5 cells was performed 48 h after the transfection with control siRNA (sc-siRNA) or PAX2 specific siRNA (PAX2-siRNA). ***P<0.001 considered statistically significant compared to control siRNA transfected cells (sc-siRNA). (**D**) The invasive capacity of SkMel5 cells was analyzed 48 h after the transfection of contol (sc-siRNA) or PAX2 siRNA (PAX2-siRNA) in an invasion assay as described under material and methods ***P<0.001 considered statistically significant compared to control siRNA transfected cells (sc-siRNA).

In the progression of melanoma the anchorage independent cell growth of melanoma cells is a crucial point for the dissemination of melanoma cells to other sides of the body [18]. To evaluate the role of PAX2 in the anchorage-dependent and -independent cell growth, PAX2 expression in SkMel5 cells was downregulated by PAX2 specific siRNA. Importantly, the downregulation of PAX2 completely inhibited the anchorage-independent cell growth of SkMel5 melanoma cells (Fig. 6A). In contrast, the PAX2 downregulation in SkMel5 cells did not lead to a complete inhibition of the anchorage dependent cell growth, but significantly reduced the proliferation of SkMel5 cells (Fig. 6B). As PAX2 is able to regulate ADAM10 expression, these data are in line with our former study where we could demonstrate that the downregulation of ADAM10 reduces the anchorage independent cell growth [17]. To determine the role of PAX2 in the migration and invasion of melanoma cells we performed migration and invasion assays of SkMel5 cells after the knockdown of PAX2. Importantly, the downregulation of PAX2 led to a significant reduction of the migratory (Fig. 6C) and invasive capacity (Fig. 6D) of melanoma cells.

### Downregulation of PAX2 in melanoma cells abrogates the chemoresistance against cisplatin

**Figure 7:**
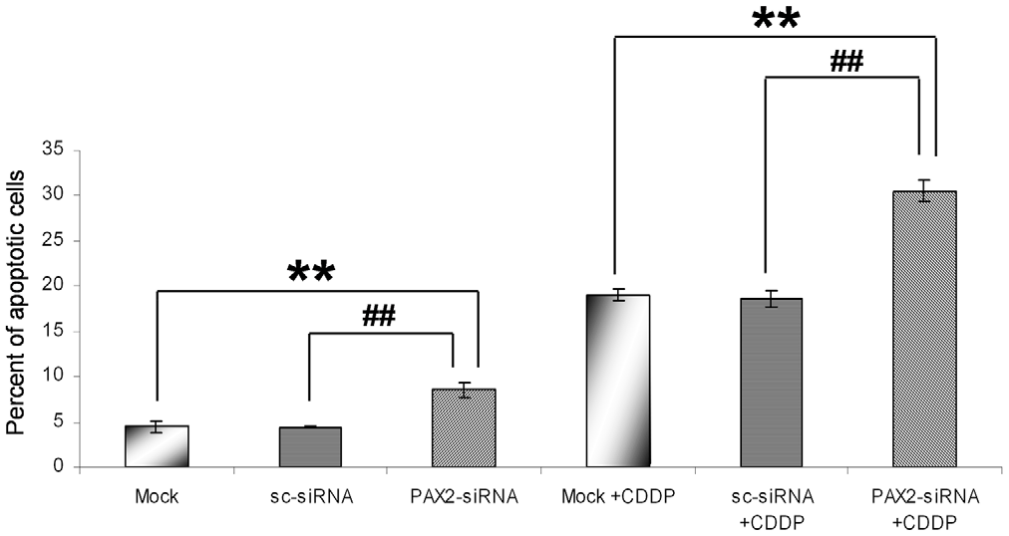
Downregulation of PAX2 abrogates chemoresistance of melanoma cells against cisplatin. 48 hours after siRNA transfection, SkMel5 cells were left untreated or treated for 24 hours with 40 µM cisplatin (CDDP). Melanoma cells were collected and analyzed with cell-cycle analysis as described under material and methods. In the graph the percentage of cells in sub-G1 phase (apoptotic cells) after the different treatments is shown. 3 independent experiments have been performed and statistical analysis was performed as described in material and methods. **P<0.01 considered statistically significant compared to Mock or Mock and cisplatin (CDDP) treated cells **##**P<0.01 considered statistically significant compared to scrambled-siRNA transfected or scrambled-siRNA transfected and cisplatin (CDDP) treated cells.

Melanoma cells are very resistant against chemotherapy and only few melanoma patients show response rates against chemotherapeutic reagents [19]. PAX2 expression has been shown to be involved in cancer cell survival [15]. To investigate if PAX2 is involved in the chemoresistance of melanoma cells, we downregulated PAX2 protein expression in melanoma cells and treated the cells with the chemotherapeutic reagent cisplatin. Interestingly, the downregulation of PAX2 alone increased significantly the number of apoptotic melanoma cells (Fig. 7). In addition, compared to control siRNA transfected melanoma cells, PAX2 downregulated melanoma cells showed a significant induction of apoptosis after the treatment with cisplatin (Fig. 7). In summary, we can conclude that PAX2 expression is involved in melanoma cell survival.

## Discussion

The PAX family of transcription factors consists of 9 members, which play crucial roles during normal development and carcinogenesis [20]. In melanocytes and melanoma it has been shown, that PAX3 is critical for the normal development of melanocytes, but plays also important roles during melanoma progression (reviewed in [21]). To investigate the role of PAX2 in melanoma development, we analyzed its expression in normal melanocytes and melanoma cells and in tissue samples of benign nevi and melanoma. Furthermore with in vitro assays we determined the role of PAX2 in melanoma progression. We found: 1) Weak PAX2 expression in normal melanocytes and increased expression in melanoma cells. 2) PAX2 was involved in the regulation of ADAM10, a transmembrane protease, which was recently found from our group to be upregulated in melanoma metastasis [17]. 3) The downregulation of PAX2 reduced the anchorage dependent and independent cell growth of melanoma cells. 4) PAX2 was involved in the migration and invasion of melanoma cells. 5) PAX2 mediated melanoma cell survival against therapeutic reagents like cisplatin. Taken all our results together, we assume that PAX2 represents an interesting new therapeutic target molecule for the treatment of patients with melanoma or melanoma metastasis. The inhibition of PAX2 in melanoma cells could reduce the proliferation, migration, invasion and chemoresistance of melanoma cells. In this context our novel and important finding that PAX2 can regulate ADAM10 expression could play a major role in the above mentioned tumor promoting functions of PAX2 in melanoma. ADAM10 belongs to the ADAM family, which cleave transmembrane proteins like growth factors, cytokines, chemokines and adhesion molecules [22]. It is known, that the soluble forms of the cleaved proteins can bind to receptors on other cancer cells and thereby induce the proliferation, migration or invasion of cancer cells [23]. In our previous study we demonstrated that ADAM10 is involved in the shedding of L1-CAM [17], a neural cell adhesion molecule known to be overexpressed in different types of cancer including melanoma [24]. L1-CAM expression in melanoma cells mediates resistance against chemotherapeutic reagents [17] and soluble L1-CAM can induce tumor cell proliferation, migration and invasion [24]. Therefore the downregulation of ADAM10 by PAX2 siRNA in melanoma cells will inhibit the production of soluble L1-CAM and therefore will inhibit the tumor-promoting function of soluble L1-CAM during melanoma progression. Another important aspect of our study is that the downregulation of PAX2 in melanoma cells abrogated the chemoresistance of melanoma cells against cisplatin. The mechanism for PAX2-mediated protection from cell death is unknown, although it has been shown that the closely related PAX8 was reported to transcriptionally activate the anti-apoptotic protein BCL2 [25]. Therefore further experiments have to be performed to identify proteins which can be regulated through PAX2 and are responsible for the chemoresistance of melanoma cells against therapeutic reagents like cisplatin.

An interesting future topic of our research will be to identify factors which are involved in the regulation of PAX2 in melanoma cells. In renal cell carcinoma it has been shown that the loss of VHL and hypoxia can upregulate PAX2 expression [26].Therefore, determining factors and their signalling pathways which are involved in the progression of melanoma which can upregulate PAX2 expression, may identify new therapeutic target proteins for the treatment of melanoma patients.

In tissue samples of benign nevi and melanoma we identified PAX2 expression in nucleoli of melanocytes and melanoma cells. Interestingly, nucleoli are dramatically modified in many human cancers. The role of the nucleolus in tumorigenesis is highlighted by the regulation of the powerful tumor supressor gene p53. It has been shown that the retention of MDM2, a key regulator of p53, in the nucleolus leads to an accumulation of p53 and the activation of p53 dependent pathways [27]. Importantly, PAX2, PAX5 and PAX8 are able to repress p53 [28]. Further experiments have to be performed to demonstrate that PAX2 can regulate p53 expression in melanoma cells and that the downregulation of PAX2 may activate p53 dependent pathways which are involved in mediating cell survival in melanoma cells. In addition, it has been shown in prostate cancer, that the inhibition of PAX2 resulted in cell death independent of p53, demonstrating that additional tumor supressors or cell death pathways are inhibited by PAX2 in prostate cancer.

In summary, our data clearly demonstrate that PAX2 regulates ADAM10 expression in melanoma cells and to our opinion PAX2 represents a new interesting therapeutic target molecule in melanoma. Further experiments in rodents will clarify, if the inhibition of PAX2 in melanoma cells will lead to a reduced melanoma growth in vivo and the development of small molecule inhibitors against PAX2 may represent a potential therapeutic option for the treatment of melanoma patients.

## Materials and Methods

### Antibodies

The ADAM10 antibody for immunofluorescence analysis was purchased from Diaclone (Besancon, France), the ADAM10 antibody for Western Blot analysis from Chemicon (Schwalbach, Germany). The PAX2 antibodies for westernblot analysis were obtained from Abcam (Cambridge, United Kingdom), for immunohistochemistry and immunofluorescence analysis from Epitomics (California, USA). The β-actin antibody for Western Blot analysis was purchased from Sigma-Aldrich (Taufkirchen, Germany). The antibodies S100 (melonocyte marker) was ordered from Santa Cruz (Heidelberg, Germany), CD31 was obtained from Dako (Hamburg, Germany).

### Human tissue samples

Slides for immune staining were prepared from excision material stored at the histology laboratory, Department of Dermatology, Clinic of the Goethe University, from a total of 13 patients. In 5 cases, the diagnosis was “naevus cell neavus”, in the 8 other cases, the diagnosis of primary malignant melanoma had been established. Tissue sections were obtained from the tissue bank of the histology laboratory , Department of Dermatology, university hospital in Frankfurt am Main (Germany).

### Immunohistochemistry

All specimens were fixed in 4% formaline (pH 7.4), embedded in paraffin followed by cutting with a microtome (3 µm thickness) and placing on SuperFrost Plus slides (Microm International, Walldorf, Germany). For immunohistochemistry, the following antibody was used: monoclonal rabbit IgG anti-human PAX2 (Epitomics, California, USA). The slides were deparaffinized in xylol for 20 minutes and then rehydrated in descending series of ethanol (100%, 100%, 96%, 96%, 70%, and 70%). For antigen retrieval the slides were boiled in citrate buffer (pH 6.0) for 40 min, and then allowed to cool down for 15 min. After washing with PBS buffer the endogenous peroxidase was blocked with H_2_O_2_ for 15 min at room temperature. After washing in PBS the slides were incubated with the antibody against PAX2 (dilution 1∶100) for 60 min at room temperature and washed in PBS again. The secondary antibody was incubated for 20 min at room temperature and after washing the slides in PBS the biotin streptavidine label was incubated for 20 min at room temperature. A detection kit including horseradish peroxidase and diaminobenzidine as chromogene was applied for 5 min (DCS, Hamburg, Germany). Counterstaining was performed with hematoxilin for 6 min.

### Cell culture

The human keratinocyte cell line HaCaT and the melanoma cell lines MeWo and Sk Mel 5 were provided from Prof. Jörg Reichrath (Department of Dermatology, The Saarland University Hospital, Homburg/Saar, Germany). The melanoma cell lines A375, IPC298, G361, NW1539 were a kind gift from Dr. Claudia Bürger (Department of Dermatology, Goethe University hospital) and human melanocytes (Mel43) were isolated as described elsewhere [29].

### siRNA transfection

For downregulation of endogenous PAX2 expression the following siRNA duplexes (MWG Biotech AG, Ebersberg, Germany) were used: PAX2-siRNA1: 5′-GAA GUC AAG UCG AGU CUA U-3′, PAX2-siRNA2:5′AUC UUC AUC ACG UUU CCU CCC CC-3′. As a negative control unspecific scrambled siRNA duplexes (5′-AGG UAG UGU AAU CGC CUU GTT-3′) were used. 24 hours before the transfection 1×10^5^ cells were seeded in 6-well plates. Transfection of siRNA was carried out using Oligofectamine (InVitrogen, Karlsruhe, Germany) and 10 nM siRNA duplex (MWG Biotech AG, Ebersberg, Germany) per well. Transfection was performed as previously described [30]. Specific silencing of targeted genes was confirmed by at least two independent experiments.

### Western Blot Analysis

Cell extracts were prepared and processed as described recently [31] at times indicated. Western Blot membranes were incubated with a rabbit antibody against human ADAM10 in a dilution of 1∶2000 (Calbiochem, Darmstadt, Germany) in 2% non-fat dry milk dissolved in TTBS (20 mM Tris, 150 mM NaCl pH 7.5, 0.1% Tween 20). Blots were developed using the ECL system (AmershamPharmacia, Buckingshamshire, UK). To confirm equal loading, blots were reprobed with a β-actin antibody (Sigma, Deisenhofen, Germany).

### Chromatin immunoprecipitation (ChIP) assay

The ChIP-IT Express kit (Active Motif, Rixensart, Belgium) was used to perform the ChIP assay. Four dishes of Sk-Mel5 cells with 1×10^6^ cells/dish were incubated for 10 min with 1% formaldehyde, followed by glycine stop solution. Afterwards, cells were harvested, centrifuged and resuspended in lysis buffer. After lysis and homogenization in a douncer, nuclei were collected by centrifugation and resuspended in shearing buffer. Enzymatic shearing cocktail was added for 10 min to digest DNA in fragments with sizes ranging from 200–500 bp. After centrifugation, 10 µl of the sheared chromatin was used as an input control. The immunoprecipitation was performed overnight using a head to head rotator. The incubation mixture contained 50 µl sheared chromatin, 100 µl protein G magnetic beads, and 3 µg PAX2 antibodies (Abnova, Heidelberg, Germany). After washing and elution, the samples were reverse cross-linked and treated with ribonuclease A and proteinase K. The DNA and the sheared chromatin input were used directly for PCR. The primer pairs 5′-GCG CGT CAC GTG GTG AGG AA-3′ and 5′-CCC TGG CAG GAG AAA CGG CG-3′, was designed to amplify a 207-bp product of the human ADAM10 promoter which contains the PAX2 binding site (Sequence: NM_001110.2).

### Fluorescence microscopy

Cells were grown on coverslips and fixed with 4% paraformaldehyde/PBS. After washing the cells with PBS, cells were permeabilized and blocked with 0.1% Triton X-100/PBS containing 5% BSA. The anti-human ADAM10 ectodomain antibody (1∶200 dilution) or PAX2 antibody (1∶100 dilution) was incubated for 1 hour at room temperature. Following 3 times washing, bound antibodies were deteced by Alexa 488 conjugated goat anti-mouse or Cy3 conjugated goat anti-mouse (Molecular Probes, Karlsruhe, Germany) secondary antibodies. Following PBS-washing nuclei were stained with 4′6-diamidino-2-phenylindole (DAPI, Sigma, Munich, Germany) and cells were mounted in Fluoromount-G™ (Biozol, Eching, Germany) and examined by fluorescence microscopy (Keyence, Neu-Isenburg, Germany) or with an LSM 510 Meta confocal laser-scanning microscope (Carl Zeiss, Jena, Germany). Quantification of fluorescence intensity was performed using software from Zeiss image program. All fluorescence images were taken under identical conditions.

### Immunofluorescence analysis of tissue sections

For immunofluorescence analysis, tissue sections were deparaffinized as described under immunohistochemistry. Antigen retrieval was performed incubating the tissue sections for 20 min in 0.01 M sodium citrate buffer, pH 6.0 in a microwave oven (500 W). After incubation with blocking buffer (0.1% Triton X-100/PBS containing 1% BSA and 10% horse serum) for 1 h, tissue sections were incubated for two hours at 37°C, than overnight at 4°C with the first antibodies (diluted in 1% BSA/10% horse serum/PBS/0.1% Triton X-100) as indicated. Following washing, bound antibodies were detected by Alexa 488 conjugated goat anti-mouse (Molecular Probes, Karlsruhe, Germany) or goat anti-rabbit Cy3 (Molecular Probes, Karlsruhe, Germany) secondary antibodies. Nuclei were stained with 4′,6-diamidino-2-phenylindole (DAPI, Sigma, Deisenhofen, Germany) and slides were mounted in Fluoromount G (Southern Biotechm, Birmingham, USA). Evaluation was performed by fluorescence microscopy and analyzed with an LSM 510 Meta confocal laser-scanning microscope (Carl Zeiss, Jena, Germany).

### Proliferation assay

24 hours after the transfection of siRNAs the MeWo cells or SK Mel 5 cells were seeded into 96 well plates. For evaluation of the anchorage-independent cell growth, plates were coated with poly-2-hydroxyethyl methacrylate (Sigma, Deisenhofen, Germany). Cell growth was monitored after 24, 48 and 72 hours using a CellTiter 96® Non-Radioactive Cell Proliferation Assay (Promega, Mannheim, Germany). The formation of formazan through cleavage of the tetrazolium salt MTT in metabolically active cells was measured at the absorbance of 570 nm using a spectrophotometer. Each assay was performed in triplicates and repeated at least 3 times. Data are presented by means ± SD. Statistical and significant differences were determined by ANOVA with post-hoc analysis.

### Cell cycle analysis

Cells were seeded in 6 well plates and transfected with siRNA as described before. 48 hours after siRNA transfection, cells were left untreated or treated with 40 µM cisplatin (CDDP). Cells were trypsinized, washed in PBS, and incubated overnight at 4°C in 1 ml hypotonic solution containing 50 µg/ml propidium iodide, 0.1% sodium citrate, 0.1% Triton X-100 and 20 µg/ml DNAse-free RNAse A. Cells were analyzed with flow cytometry in linear mode. Results were expressed as percentage of elements detected in the different phases of the cell cycle, namely sub-G_1_ –peak (apoptosis), G_0_/G_1_ (no DNA synthesis) S (active DNA synthesis), G_2_ (premitosis) and M (mitosis). Statistical and significant differences were determined using the Student's T-Test.

### Cell migration and invasion assay

The effect on cell migration was measured as the ability of cells to migrate through Transwell filters (Corning, Amsterdam, Netherlands, 6.5 mm diameter, 5 µm pore size). Transwell filters were coated with fibronectin (10 µg/ml in PBS) or matrigel (diluted 1∶4) for 90 min before adding the cells. At 48 hours after the siRNA transfection, cells were detached by trypsinization and 1×10^5^ cells were seeded into transwell filters in 100 µl starvation medium. 500 µl growth medium was placed in the lower compartment, and the cells were left to migrate for 16–20 hours. Non migrated cells were removed by a cotton swab, the transmigrated cells at the backside of the filter were stained with crystal violet solution as described [32]. The eluted dye was measured at 595 nm in an ELISA reader. Each experiment was performed in triplicates and repeated at least thrice. Data are presented by means±SD. Statistical and significant differences were determined using Student's t-test.
